# The evolved nest, oxytocin functioning, and prosocial development

**DOI:** 10.3389/fpsyg.2023.1113944

**Published:** 2023-06-22

**Authors:** Mary S. Tarsha, Darcia Narvaez

**Affiliations:** Department of Psychology, Kroc Institute for International Peace Research, University of Notre Dame, Notre Dame, IN, United States

**Keywords:** oxytocin, early life experience, empathy, evolved nest, parenting, prosocial

## Abstract

Prosociality, orientation to attuned, empathic relationships, is built from the ground up, through supportive care in early life that fosters healthy neurobiological structures that shape behavior. Numerous social and environmental factors within early life have been identified as critical variables influencing child physiological and psychological outcomes indicating a growing need to synthesize which factors are the most influential. To address this gap, we examined the influence of early life experiences according to the evolved developmental niche or *evolved nest* and its influence on child neurobiological and sociomoral outcomes, specifically, the oxytocinergic system and prosociality, respectively. To-date, this is the first review to utilize the evolved nest framework as an investigatory lens to probe connections between early life experience and child neurobiological and sociomoral outcomes. The evolved nest is comprised of characteristics over 30 million years old and is organized to meet a child’s basic needs as they mature. Converging evidence indicates that humanity’s evolved nest meets the needs of a rapidly developing brain, optimizing normal development. The evolved nest for young children includes soothing perinatal experiences, breastfeeding, positive touch, responsive care, multiple allomothers, self-directed play, social embeddedness, and nature immersion. We examined what is known about the effects of each evolved nest component on oxytocinergic functioning, a critical neurobiological building block for pro-sociomorality. We also examined the effects of the evolved nest on prosociality generally. We reviewed empirical studies from human and animal research, meta-analyses and theoretical articles. The review suggests that evolved nest components influence oxytocinergic functioning in parents and children and help form the foundations for prosociality. Future research and policy should consider the importance of the first years of life in programming the neuroendocrine system that undergirds wellbeing and prosociality. Complex, interaction effects among evolved nest components as well as among physiological and sociomoral processes need to be studied. The most sensible framework for examining what builds and enhances prosociality may be the millions-year-old evolved nest.

## Introduction

Individual and societal thriving depend upon sociomoral capacities, such as empathy, and prosociality, an orientation to attuned, empathic relationships. Though western traditions have largely emphasized top-down socialization of sociomorality (i.e., verbal teaching and coercion; Kant, 1964), emerging research in developmental neurobiology demonstrates that prosocial capacities are rooted in neurophysiological structures that are shaped in early life through supportive care (Atzil et al., 2018). This is because the brain is most plastic in early life, experience expectant, and shaped by social experience (Coulombe et al., 2019; Phua et al., 2020). We address humanity’s evolved system for raising the young, the *evolved nest* (Narvaez, 2014), focusing on one biological aspect known to correlate with empathy and prosociality, the neuropeptide oxytocin, whose function is influenced by early life experience.

### Oxytocin: critical neuropeptide for health and prosociality

Oxytocin is a powerful neuropeptide, a pleiotropic hormone of critical importance for social bonding and health across the lifespan. Consisting of nine amino acids, oxytocin is produced in the hypothalamus and plays a role in integrative functions (coordination of physiology and behavior) such as birth, lactation, maternal behavior, and romantic bonding (Carter, 2014). The complex pathways oxytocin travels contribute to its widespread function, allowing it to operate not only as a hormone, but also as a neurotransmitter and a paracrine substance (cell-to-cell communicator), enabling it to play a role in regulating numerous physiological systems including reproductive, immune, and autonomic and central nervous systems (Carter et al., 2020). It also supports the functioning of the vagus nerve, a major branch of the parasympathetic nervous system that innervates all the major organs of the body to maintain health (Porges, 2007). The well-functioning vagus is associated with a sense of safety, contributing to capacities for social closeness and sensitivity within caregiver-infant interactions as well as in adult romantic relationships (Porges, 1998, 2003). Oxytocin supports patterns of growth, resilience and healing by increasing anti-inflammatory and antioxidant capabilities and supporting stress coping (Carter et al., 2020). In early life, parental oxytocin release combined with behavioral synchrony between parent and child influence child sociality (Feldman, 2015).

Research into oxytocin has taken two approaches. Some studies involve *exogenous* oxytocin, the administration of the neuropeptide in experimental protocols, whereas other studies investigate *endogenous* (embodied or made naturally within the body) oxytocin. Both exogenous and endogenous oxytocin are closely associated with social behavior which makes sense in light of the broad range of oxytocin receptors present throughout the brain. Receptors are specifically expressed in the social brain network, comprised of the prefrontal cortex, cingulate cortex, and amygdala, areas of the brain that are involved in reproduction, maternal behavior, affiliation, and attachment (Nishizato et al., 2017).

*Exogenous* oxytocin, typically administered nasally, decreases threat reactivity by dampening amygdala reactivity to emotional stimuli, making it useful in treating social anxiety (Jones et al., 2022). Exogenous oxytocin also boosts empathy and prosocial behavior in adults (Domes et al., 2007; Kanat et al., 2015). However, the relationship between prosociality and exogenous oxytocin is more complicated than simply increasing positive outcomes. Depending upon personal history, including attachment style, it can increase antisocial behaviors such as gloating, envy, out-group hostility and ethnocentrism (Keltner et al., 2014). Further, sociomoral outcomes vary. For example, one study found that exogenous oxytocin enhanced *emotional* empathy (feeling with) but not *cognitive* empathy (perspective taking; Hurlemann et al., 2010), and another found that it increased compassion-focused imagery but only in individuals with higher attachment security (Rockliff et al., 2011).

*Endogenous* oxytocin is also associated with prosociality, including compassionate behavior (Eisenberg and Eggum, 2008). In a convenience sample of adults ranging from 18 to 99, Zak et al. (2022) found correlations of oxytocin release with prosocial behaviors (donations to charity, volunteering). Oxytocin release increased with age and moderated the impact of age on prosocial behaviors. However, the association between endogenous oxytocin and prosociality may not be a direct linear association but may depend upon context. For example, increased endogenous oxytocin levels within the parent-infant relationship promote affiliative and nurturant behaviors such as tend-*befriend* behavior but when engaged in in-group vs. out-group interactions, oxytocin supports affiliative tend-*defend* motivations and behavior (Taylor et al., 2006; Campbell, 2008; Crockford et al., 2014). Variations in oxytocin release and social behavior may be attributed to its widespread interactions with other neuroendocrine systems (Crockford et al., 2014).

Advances in developmental psychobiological research demonstrate that dynamic interactions in early life—the interplay between the child and the caregiving environment—shape biological processes, including oxytocinergic functioning (Lomanowska et al., 2017). For example, when optimally operative, oxytocin serves as a protective mechanism against stress and adversity, supporting resilience and adaptation at every age (Smeets, 2010; Sharma et al., 2020). However, the degree to which the oxytocinergic system develops optimally depends upon experience, specifically, early relationships (Carter et al., 2009; Köhler-Dauner et al., 2019). Consequently, the last two decades of developmental psychobiological research has focused on examining a wide range of environmental influences including the quality and nature of caregiving on child outcomes (Calkins, 2011; Veenema, 2012; Sandi and Haller, 2015; Onaka and Takayanagi, 2021).

The focus of this review was to address what is known regarding the role of early experience on oxytocinergic functioning and its relation to prosocial capacities. The specific sociomoral capacities targeted were empathy and prosociality, especially as they relate to oxytocinergic functioning (e.g., Apter-Levy et al., 2013; Carter et al., 2017). It was expected that studies would show that the developmental trajectory of oxytocinergic functioning depends upon and is influenced by early care consistent with our species’ developmental system, the *evolved nest*.

### The evolved nest

Humanity’s *evolved nest* emerged from the social mammalian line which is over 30 million years old (Konner, 2005). Like every animal’s developmental system, the *evolved nest* is organized to meet the basic needs of the young as they mature (Gottlieb, 2002). Also named the hunter-gatherer childhood model (Konner, 2005), *evolved nest* provision is still apparent in today’s nomadic foraging communities, the type of society in which humanity spent at least 95% of its existence, which we call our ancestral context (Lee and Daly, 2005; Fry, 2006). Over the course of human evolution, a large social brain emerged, with distinctive capacities from those of chimpanzees, accompanied by intense child raising (Burkart et al., 2009; Hrdy, 2009a). Because they are highly immature at birth with only about a quarter of adult brain volume, resembling fetuses of other animals until about 18 months of age, humans are particularly dependent on their *evolved nest* (Trevathan, 2011). Human infants expect an ‘external womb’ experience during the early months of life, which facilitates healthy development (Montagu, 1986). Well-nurtured, species-normal organisms exhibit biological self-regulation, good health, and fittedness in the ecological and social environment in which they exist, operating effectively with conspecifics and other organisms. Humanity’s *evolved nest* is designed to prepare a child for self-regulation, good health, social and ecological fittedness and, theoretically, enhanced sociomoral capacities of prosociality and empathy, required for adaptation (Narvaez, 2014).

Provisioned by a community and not simply by mothers, the *evolved nest* for young children includes (Hewlett and Lamb, 2005; Narvaez and Bradshaw, 2023): (1) soothing perinatal experiences; (2) breastfeeding on request for several years; (3) nearly constant positive touch; (4) appropriate responses to keep baby optimally aroused; (5) multiple allomothers (care by responsive individuals other than mothers such as fathers and grandmothers); (6) multiage self-directed free play; and (7) social embeddedness, with a positive, welcoming social climate, and (8) nature immersion. Ethnographic evidence suggests that communities that provide the *evolved nest*—nomadic foragers—exhibit highly prosocial, compassionate, generous, caring, and egalitarian behavior (Wolff, 2001; Ingold, 2005; Fry and Souillac, 2013; Narvaez et al., 2013c; Narvaez, 2019; Topa and Narvaez, 2022). Thus, it has been proposed that the *evolved nest* may be necessary for the development of species-typical sociomoral capacities (Narvaez, 2014). Converging evidence from developmental psychology, neuroscience, evolutionary biology, and epigenetics suggests that *evolved nest* components may be critical variables in shaping oxytocinergic functioning (e.g., Schore, 2003; Kim et al., 2011; Carter and Porges, 2013; Champagne, 2018). We examined empirical evidence regarding the relation of *evolved nest* components to oxytocin system functioning. Oxytocin system development may be part of the physiological foundations for humanity’s social fittedness.

## The review of direct and indirect effects

In this review, we note what is species-normal in humanity’s *evolved nest* context. We examine what is known regarding the specific effects on oxytocinergic functioning of each *evolved nest* component (perinatal factors, breastfeeding, touch, responsiveness, alloparents, free play, social embeddedness or positive climate, and nature connection). Across the numerous empirical and theoretical studies reviewed, the terms “adaptive, “optimal,” and “regulated” are employed to refer to behavioral and physiological processes that are advantageous. That is, oxytocin levels considered adaptive or regulated are discussed in terms of specific empirical contexts (Porges, 2007). For example, if a stress condition is employed, researchers discuss adaptive responses in terms of self-regulatory processes and successful completion of tasks.

**Figure 1:**
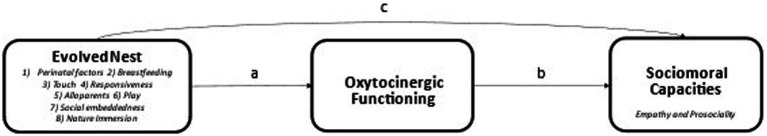
Overview of the review: examining the role of the evolved nest on neurobiological functioning that gives rise to sociomoral capacities.

Regarding scope, each *evolved nest* component is discussed in relation to empirical evidence available for oxytocinergic functioning (see Figure 1, Path a). Next, the relation between the *evolved nest* component and empathy and prosociality is discussed, again, in terms of empirical evidence of oxytocin functioning (Figure 1, Path b). We call this path the indirect path because it examines how *evolved nest* components relate to empathy and prosociality through oxytocinergic functioning (Paths a and b). We also examine connections between evolved nest components and empathy and prosociality, not taking into account oxytocin (Figure 1, Path c). We call this path the direct path. When little information is available regarding a specific *evolved nest* component and empathy or prosociality, correlates of prosociality are employed. Lastly, gaps in the literature regarding the *evolved nest*, oxytocin and prosociality are highlighted, noting areas for future research.

### Soothing perinatal experiences

Within the *evolved nest* context, the mother is supported by other women and community members during pregnancy, at birth and postnatally. Birth follows the natural rhythms of the mother and child, which allows optimal physiological and psychological processes to prepare the mother’s and infant’s bodies for labor and birth (Trevathan and McKenna, 1994; Trevathan, 2013). For the mother, physiological preparedness before the onset of labor and birth includes low social stress and a rise in oxytocin as well as other hormones (e.g., estrogen, beta-endorphins and prolactin; Buckley, 2015). For infants, who vary in womb residence by about 55 days, determining their own birth date is more likely to enable physiological preparedness such as maturation of lungs and other organs to facilitate adjustment to life outside the womb (Trevathan, 2011, 2013). At birth, increases in catecholamines promote breathing, glucose production and heart regulation. *Evolved nest* provision during the perinatal period means the child experiences supportive, comforting, and soothing care and harsh, intrusive, and painful procedures are avoided. There is little interference in natural processes. Support of the dyad provides opportunity for physiologically-driven maternal–infant bonding, along with breastfeeding success, infant adaptation, and a healthy start (Buckley, 2015).

#### Perinatal experiences and oxytocin

The effects of endogenous oxytocin during birth are specific—e.g., inducing uterine contractions—and broad—impacting numerous neurobiological systems in both mother and infant. Mice studies demonstrate that during birth, oxytocin operates through a series of pathways that overlap with numerous other hormonal systems such as cortisol and corticotrophin-releasing hormone (Gross et al., 2000; Ratajczak and Muglia, 2008). Similarly, in humans, oxytocin is part of a much larger cascade of neuroendocrine pathways that overlap and interact with each other to initiate and sustain successful childbirth. Disrupting experiences, such as interference or complications during birth, potentially alter this complex cascade of neurobiological pathways, including oxytocin release. For example, when comparing birth types and measuring oxytocin levels immediately before delivery, vaginal births are associated with the highest level of maternal venous oxytocin concentrations whereas cesarean section births have the lowest levels (Marchini et al., 1988; Moberg and Prime, 2013). The same oxytocin patterns (assessed via umbilical cord) are observed in corresponding infants.

Neurobiological factors that contribute to the onset of the birth process involve a complex interaction among mother, infant, and placenta (Kenkel et al., 2014). Maternal oxytocin increases throughout pregnancy to facilitate calmness and preparedness for childbearing. During birth, maternal oxytocin is released both centrally (in the brain) as well as induced through activation of afferent sensory nerve fibers in the mother’s pelvis (Uvnäs-Moberg et al., 2020b). When the infant’s head exerts pressure on the cervix and vaginal wall, oxytocin is released into the mother, an effect known as the Fergusson reflex. Maternal pulses of oxytocin in the brain released during the childbearing process activates her reward system, decreasing pain, fear, and stress, and increasing bonding to the newborn (Buckley, 2015). In addition, the actual process of childbirth itself increases maternal oxytocin receptor expression (Broad et al., 1999), thought to promote breastfeeding and healthy attachment.

For the fetus before birth, oxytocin is released into fetal circulation from the fetal brain (via the posterior pituitary gland) bringing about multiple physiological benefits (Strathearn, 2011). These benefits include preparing the infant for birth and the extrauterine environment, and triggering changes to fetal gamma-aminobutyric acid (GABA), a neuroinhibitory transmitter that functions to reduce excitability in neurons, which is critical for healthy neuronal development (Tyzio et al., 2006; Khazipov et al., 2008). Prior to delivery, GABA functions as an *excitatory neurotransmitter* in the fetus, rather than its usual role as a prototypical *inhibitory signal* in the mature brain. Oxytocin supports the critical transition from excitatory to inhibitory functioning, catalyzing neurons to undergo electrophysical changes, critical for the infant’s survival because the oxytocin-dependent GABA transition protects the fetal brain from hypoxic conditions that otherwise might take place during parturition. In addition, the oxytocin-induced GABA transition also acts as an analgesic, reducing polarization of GABA on nociceptive (pain) neurons. Thus, oxytocin not only initiates birth through maternal uterine contraction, but also changes the infant’s GABA neuromodulators from inhibitory to excitatory which later protects the newborn from both pain and hypoxia.

What about synthetic (exogenous) oxytocin given to pregnant women to induce labor? Perinatal exogenous oxytocin is linked to detrimental outcomes such as neonatal jaundice, decreased breastfeeding and interference with mother-infant bonding (Schiefenhövel and Trevathan, 2019; Trevathan and Rosenberg, 2019). In addition, higher synthetic oxytocin administration during labor is associated with an increase in maternal depression, anxiety, and somatization symptoms 2 months after giving birth, even controlling for perinatal posttraumatic stress related to childbirth experiences (Gu et al., 2016). Consequently, there is a growing interest in investigating the routine practice of administering synthetic oxytocin during labor. However, some evidence indicates that oxytocin administration can curb the effects, not only of dangerous, prolonged labor but under some conditions, postpartum hemorrhage (Kenkel et al., 2014). Nonetheless, its routine use is questionable considering the potential long-term adverse effects (Harris and Carter, 2013).

Considering the growing consensus regarding the potential adverse effects of routine oxytocin administration during childbirth, there is increased interest in facilitating endogenous oxytocin through natural methods that were common in our ancestral context (Trevathan, 2013). These include physiological manipulations such as tactile stimulation of sensory nerves (e.g., nipples), gentle touch, and calming, supportive social interactions (Olza et al., 2020). The latter can be implemented by the woman’s partner or midwife to reduce stress, pain, and anxiety. However, few studies have been conducted that measure the rise of oxytocin during such body-centered interventions during childbirth (Tarsha et al., 2020). Following the natural rhythms of the mother–child dyad in the perinatal period may also provide rich data.

#### Perinatal experiences and prosociality

Regarding prosociality, during pregnancy mothers with higher *endogenous* oxytocin show higher scores on theory of mind, a construct closely associated with empathy, even after controlling for parity, maternal education, prenatal psychosocial risk, and general anxiety (MacKinnon et al., 2014). Endogenous perinatal oxytocin effects are understudied, though uninterrupted natural birth practices may be associated with greater maternal empathy for the neonate because of natural oxytocin flow. More empirical evidence is available for *exogenous* perinatal oxytocin and its influence on disorders associated with deficits in empathy, as well as. increased risk for child psychiatric conditions such as bipolar disorder and cognitive impairment (Freedman et al., 2015). However, a recent meta-analysis by Lønfeldt et al. (2019) concluded that administered perinatal oxytocin’s relationship to psychiatric conditions is premature and more research is needed to accurately assess its influence on child development.

### Breastfeeding

Within the *evolved nest* context, breastfeeding takes place on-request, following the subtle and overt signals of the infant, and begins immediately following birth, persisting anywhere from 2.5 to 5 years or longer, with an average age of weaning around 4 years (Hrdy, 2009a). Unfortunately, in the United States, only 25% of women exclusively breastfeed their infants at 6 months of age with 34% at 1 year (Louis-Jacques and Alison Stuebe, 2018). Most studies comparing breastfeeding with formula-feeding examine only 3 months’ worth of feeding, thus findings do not encompass species-typical practice. Moreover, studies typically do not report on whether the infant is in charge of feeding, or how often or whether the infant’s signals to feed are always fulfilled.

Breast milk is the most beneficial source of nutrition for an infant, but breastfeeding involves much more than the delivery of nutrition. Comprised of water (87%), proteins (1.0%), fat (3.8%), and lactose (7%), breast milk also has a host of immune cells such as microRNAs, hormones, bioactive compounds with anti-inflammatory and anti-infective properties such as cytokines, chemokines, immunoglobulins, growth factors, oligosaccharides, and antimicrobial peptides such as bacteriocin and lactoferrin (Duale et al., 2022). In comparison to infant formula, the benefits of breastfeeding—for both the mother and the infant—are numerous. For the infant, benefits encompass healthier brain development including greater neuronal myelination (Der et al., 2006; Victora et al., 2015), higher intelligence scores (Horta et al., 2015), and lower risk of infections and death (Rollins et al., 2016). Benefits for breastfeeding mothers comprise reduced stress and depressive symptoms and increased maternal–infant attachment (Krol and Grossmann, 2018), as well as long term protection against ovarian and breast cancer (Victora et al., 2016). Breastfeeding mothers also demonstrate enhanced emotion cue detection (Krol et al., 2014; Matsunaga et al., 2020).

#### Breastfeeding and oxytocin

Breastfeeding can be regarded as the final stage of labor (Labbok, 2001), providing a natural analgesic for infants (Gray et al., 2002). Oxytocin is critical for breastfeeding success due to its role in the milk ejection reflex (Erickson et al., 2020). Prolactin promotes milk synthesis and oxytocin milk release. When breastfeeding occurs, oxytocin, along with other hormones including estrogen, are released into the maternal brain, resulting in numerous physiological changes (Moberg and Prime, 2013).

Oxytocin in breastmilk, specifically colostrum which is not found in infant formula, is associated with anti-inflammatory effects in rats. More specifically, Klein et al. (2017) found that oxytocin in breast milk protected enterocytes (cells in the intestinal lining) from apoptotic cell death and supported cellular metabolism to promote enterocyte cell differentiation (cells becoming individualized for different functions). Further, oxytocin present in colostrum and breastmilk protected the intestinal villi from cellular stress. Oxytocin also downregulates inflammation processes by impeding protein translation and the promotion of autophagy (the cleaning out of damaged cells). As such, at least in rats, oxytocin in colostrum and breastmilk may be an important component of building a healthy gut microbiome and reducing inflammation.

In human mothers, release of oxytocin is catalyzed through sensory nerves in the mother’s breasts which release, on average, pulses of oxytocin every 90-s over the first 10 min of breastfeeding (Jonas et al., 2009). Within the first minute of infant suckling or stimulation with a breast pump, milk ejection takes place (Ramsay et al., 2004). During milk ejection, oxytocin is released from the magnocellular neurons of the pituitary gland’s supraoptic (SON) and paraventricular (PVN) nuclei of the hypothalamus, which also cause uterine contractions, facilitating the expulsion of the placenta. The PVN also have axons that go directly into the brain which influence activity of the hypothalamus-pituitary–adrenal axis (HPA), the autonomic nervous system including the vagus nerve, affecting general wellbeing and social interactions (Veenema and Neumann, 2008), thereby increasing maternal wellbeing.

For the infant, multiple sensory nerves are stimulated during breastfeeding through which oxytocin is released. Sensory nerves present in the infant’s oral mucosa and other parts of the infant’s face and body, including the stomach itself, are all stimulated by breastmilk and breastfeeding (Moberg and Prime, 2013). All of these sensory nerves lead directly into the PVN and SON to catalyze oxytocin release in the breastfeeding infant.

Breastfeeding has also been associated with maternal stress reduction and enhanced positive mood through oxytocinergic functioning. Surges in oxytocin during breastfeeding are known to dampen the effects of stress hormones (Cox et al., 2015). Oxytocin interacts with the HPA by lowering adrenocorticotropin hormone (ATCH) and cortisol (Uvnäs-Moberg et al., 2020a). Self-report studies confirm oxytocin’s role in reducing stress: breast-feeders reported less perceived stress than bottle-feeders, than mothers who never breastfed before, and than those who breastfed in the past but who had stopped breastfeeding (Mezzacappa and Katkin, 2002). Further, there was a difference in women’s mood before and after feeding. Breastfeeding mothers reported a decrease in *negative* mood whereas bottle-feeding mothers reported a decrease in *positive* mood. This association makes sense in light of the relationship between breastfeeding and increased maternal oxytocin levels. For example, Grewen et al. (2010) compared mothers who reported breastfeeding 90% or more of the time in comparison to mothers who reported formula feeding at least 80% of the time. Across five changing experimental conditions, breastfeeding mothers had significantly higher levels of oxytocin than bottle-feeding mothers—measured by both plasma and saliva. The neurochemical and the self-report studies provide converging evidence that breastfeeding is related to increased oxytocin levels and may play an important part in reducing maternal stress and enhancing mood, as well as facilitating the continuation of breastfeeding. Their connection may be rooted in oxytocin being released by the PVN, as outlined above, because its axons both facilitate milk let down and also connect to numerous other systems that enhance wellbeing (Veenema and Neumann, 2008).

Given the difference in maternal oxytocin levels and breast/bottle feeding, other studies have investigated the relation of feeding type, maternal mental health and oxytocin levels. Several studies found that mothers who report more anxiety and depression also demonstrate lower levels of oxytocin when breastfeeding than asymptomatic breastfeeding mothers (Stuebe et al., 2013; Cox et al., 2015; Pawluski et al., 2017). More recently, Whitley et al. (2020) found evidence to challenge this claim. Utilizing longitudinal methods, the group investigated oxytocin levels in 222 breastfeeding mothers when infants were 2 and 6 months of age and also measured antidepressant use and anxiety and depression symptoms. Maternal anxiety and depressive scores were not related to oxytocin levels at either time point. However, the group did find an interaction between antidepressant use and oxytocin levels. Mothers taking antidepressants had significantly lower oxytocin levels when breastfeeding than mothers not on medication. Given their findings, more research is needed to disentangle the relationship between maternal oxytocin, depression, anxiety, antidepressant use and breastfeeding.

Regarding *exogenous* oxytocin when given to induce labor, there are connections between maternal endogenous oxytocin levels and breastfeeding behavior. Jonas et al. (2009) found an inverse association such that as the dose of synthetic oxytocin given during labor *increased*, the level of endogenous oxytocin during breastfeeding postpartum (day 2) *decreased*. Further, women who had both administered oxytocin *and* an epidural (painkiller) had the *lowest* endogenous oxytocin levels when breastfeeding compared to other women (natural birth, or epidural but no administered oxytocin). Gu et al. (2016) found that higher doses of administered oxytocin during labor *decreased* maternal breastfeeding duration. As the dosage of administered oxytocin given during labor increased, mothers at 2 months postpartum were less likely to continue breastfeeding. Together, these findings suggest that breastfeeding increases maternal endogenous oxytocin whereas administering synthetic oxytocin during labor interferes with this association and may decrease breastfeeding duration. This also underscores the possible interrelatedness of *evolved nest* components on neurobiological processes that influence behavior: perinatal experiences, such as oxytocin administration, may influence maternal oxytocin levels later (after the infant is born), affecting subsequent breastfeeding behavior.

#### Breastfeeding and prosociality

For mothers, breastfeeding supports empathic, attuned, prosocial responses that include emotion recognition. For example, Krol et al. (2014) found that longer durations of exclusive breastfeeding were associated with faster recognition of happiness and slower recognition of anger, both responses that are associated with prosocial responsiveness. This combination of emotional detection (high emotional detection for happiness but low for anger recognition) is thought to be important for rapid responses to affiliative stimuli by also reducing the importance of threatening influences. Breastfeeding mothers show increased empathy to their own infant crying (Kim et al., 2011). Finally, a more recent study found that oxytocin was positively related with empathy and breastfeeding intention (Permatasari and Syafruddin, 2022).

For children, breastfeeding may be associated with increased empathy. Narvaez et al. (2011) found that breastfeeding length was correlated with reported empathy in three-year old children. Saarinen et al. (2020) examined the longitudinal effects of breastfeeding on adult dispositional compassion and empathy. Utilizing a large sample (*N* = 1,394), breastfeeding did not predict either sociomoral outcomes even when controlling for age, gender, socioeconomic factors and family environment. However, most participants (913) were breastfed for less than 5 months and only 36 were breastfed more than 12 months. As noted earlier, no known studies examine the average length of species-typical breastfeeding (4 years). More research is needed that pays attention to dosage and includes a more species-typical range in investigating the possible long-term effects of breastfeeding on empathy.

### Positive touch

Touch is a complex set of behaviors but can generally be divided into positive or negative categories. Positive touch experiences refer to welcomed affectionate touch, whereas we discuss negative touch as the presence of harsh touch. Within nomadic forager communities, contexts where the *evolved nest* is provided, Schiefenhövel and Trevathan (2019) noted how positive touch is abundant and provided from the first moments after birth. A short initial cry immediately after birth, as the lungs take in air for the first time, is an expected phenomenon in these societies but otherwise crying is not expected since it is a costly metabolic expenditure. Crying is mitigated in order to facilitate infant survival both in terms of energy preservation and the possible alerting of predators to the vulnerable mother and infant. Consequently, immediately after birth, the initial cry of the infant is met with an abundance of affectionate touch that includes cradling and soothing. After this, babies and young children continue to remain physically close, in contact with their mothers and others at all times, including at nighttime (Hewlett and Lamb, 2005).

As social mammals, infants and children need extensive positive (affectionate) touch for proper development and are more likely to suffer adverse outcomes if they are deprived of it (Montagu, 1986; Narvaez et al., 2019). Decades of empirical evidence (Spitz, 1945; Maitre et al., 2017) demonstrate that affectionate touch is the cornerstone of interpersonal interactions and sensory-cognitive development. Positive touch facilitates social and emotional (Feldman and Eidelman, 2004), neurobehavioral and cognitive development (Feldman and Eidelman, 2003). Frequency of maternal affectionate touch is also associated with increased social orientation in infants, which contributes to the development of the social brain (Reece et al., 2016) and body awareness (Crucianelli and Filippetti, 2020). Adverse outcomes associated with deprivation of tactile stimulation have been well documented in orphanages (e.g., Maclean, 2003). Orphans deprived of touch demonstrated adverse intellectual, physical, behavioral, and socio-emotional outcomes in addition to diminished capacities to modulate sensory experiences (Wilbarger et al., 2010).

Recent neuroimaging studies support the view that infants evolved for positive touch from the first months of life. Using diffuse optical tomography with 2-month-old infants, Jönsson et al. (2018) found that slow stroking touch (similar to emotionally valent touch or caressing), but not fast touch, yielded neurological activations similar to activations in developed, adult brains. From the earliest months of life, positive emotional or affective touch is not only encoded in the infant’s brain in a mature way, but infants are also able to discern types of touch. This suggests that infants are wired for emotional touch from the first months of life.

In addition to caressing touch, human infants may expect positive *moving* touch, which promotes brain development. A recent study demonstrated the calming effects of moving touch on an infant (Ohmura et al., 2022). At the societal level, those that keep young children ‘in arms’ and breastfeed for at least 2.5 years are more likely to be peaceful, explaining 80% of the variance among over 400 societies; no sanction of premarital sex, a touch-filled activity, explained 100% of the variance (Prescott, 1996).

#### Negative touch

Corporal punishment is a type of negative touch that has different connotations both in the vernacular as well as in different cultural settings. Following legal definitions, Gershoff (2002) defined corporal punishment as “the use of physical force with the intention of causing a child to experience pain but not injury for the purposes of correction or control of the child’s behavior” (p. 540). A wealth of literature has delineated the long-term negative consequences of corporal punishment, such as spanking, hitting, and pinching children (Fergusson et al., 2008; Grogan-Kaylor et al., 2018). Over a span of five decades, numerous cross-cultural studies, longitudinal investigations (Berlin et al., 2009; Gershoff et al., 2012), and large meta-analyses (Gershoff, 2002; Gershoff and Grogan-Kaylor, 2016) provide converging evidence regarding the harmful outcomes, which include depression or depressed mood (Fergusson et al., 2008), antisocial behavior (Straus et al., 1997), substance use, suicidal ideations (including attempts) and self-injurious behavior (Afifi et al., 2017). The meta-analysis of Gershoff and Grogan-Kaylor (2016) found no effect size difference between spanking and physical abuse, demonstrating that both share similar associations with detrimental child outcomes. This is consistent with previous findings investigating spanking alone, suggesting “spanking is empirically similar to physical and emotional abuse” (Afifi et al., 2017, p. 25).

#### Touch and oxytocin

Together with caregiver warmth and odor, positive touch induces the release of oxytocin (Uvnäs-Moberg et al., 1987), which is particularly evident in both members of the mother-infant dyad immediately after birth (Uvnäs-Moberg et al., 2015; Moberg et al., 2020). Pulses of oxytocin are released in the mother and infant postpartum when in physical contact with one another (Bigelow and Power, 2020). Matthiesen et al. (2001) observed that, similar to other mammals, newborn human infants manipulate the mother’s breast to initiate breastfeeding. In addition, a coordinated pattern of infant hand movements and suckling was identified; the repetitive breast massage by the infant stimulated a significant increase in maternal plasma oxytocin (*p* < 0.005). Skin-to-skin contact immediately following birth promoted greater oxytocin levels in fathers as well (Cong et al., 2015) demonstrating a possible biological mechanism for healthy parent-infant attachment (Levine et al., 2007). As the child develops, positive touch experiences such as hugs, back rubs, and other forms of affectionate touch continue to facilitate the release of oxytocin (Field, 2014). The short- and long-term benefits of oxytocin induced via positive touch experienced across the lifespan are numerous and include increased social bonding, wellbeing, vagal stimulation, and decreased aggression, sympathetic activation, stress reduction (decreased HPA activity), pain, and fear (Uvnäs-Moberg et al., 2020a).

Regarding negative touch and oxytocin, more studies have investigated the role of *deprivation* of touch (or low vs. high positive touch) rather than harsh touch or corporal punishment. For example, variations in maternal care are associated with differences in oxytocin receptor levels in the rat (Champagne et al., 2001). Specifically, maternal licking and grooming (LG), a form of tactile stimulation and affiliative care, is related to oxytocin receptor levels such that mothers who demonstrated higher LG have significantly higher receptor oxytocin levels. The relationship between maternal affiliative touch and maternal oxytocin levels has been observed in other mammals (monogamous and polygamous volves; Insel and Shapiro, 1992). Further, cross-fostering provides evidence that differences in oxytocin levels in mothers were dependent upon their childhood maternal care (Champagne et al., 2006). Cross-fostering involves the rearing of pups from offspring of mothers with significantly different levels of oxytocin and estrogen—the latter hormone is known to influence levels of oxytocin. When pups from mothers with low oxytocin and estrogen levels were placed in the care of mothers with high oxytocin and estrogen levels, pups developed high estrogen and oxytocin levels.

#### Touch and prosociality

In observation studies of the effects of touch on behavior, people are more responsive to requests when they are accompanied by touch, such as rating salespersons more highly or providing higher tips to wait staff (Heslin and Patterson, 1982; Joule and Gueguen, 2003; Guegen, 2004). We can assume that such behaviors are guided, at least in part, by oxytocin release, which friendly touch promotes.

Narvaez et al. (2019) conducted several studies examining connections between touch and sociomorality. In Study 1 (*n* = 156), moderate to strong correlations were found between maternal reports of touch (in infancy and touch concurrently) with their preschoolers’ sociomoral capacities, including empathy. In Study 2 (*n* = 682), using an existing longitudinal observational and interview dataset with at-risk mothers and their children in the first 3 years of life, children whose mothers were more affectionate through the years were more concerned and caring about others. Low corporal punishment and high touch predicted empathy and other sociomoral outcomes. In Study 3 (*n* = 607), adult reports of *evolved nest*-history indicated relations between positive touch and empathy. Specifically, positive touch predicted greater emotional empathy and perspective taking whereas corporal punishment predicted less. In general, across studies, more affectionate touch and less punishing touch were positively associated with wellbeing and development of sociomoral capacities.

### Responsive relational care

Providing responsive care means meeting the needs of the infant or child in the present moment, keeping infants in an optimal state of arousal—not stressed and not under aroused (Schore, 2019). Within the *evolved nest* context, children receive consistently warm, responsive care by mothers and other caregivers (see section on alloparents), starting from their earliest moments of life throughout childhood (Narvaez et al., 2019).

According to western science, responsive care requires the implementation of consistent and accurate detection and recognition of children’s overt signals, especially in times when the infant or child is distressed (Szymanska et al., 2017). Responsivity includes mutual gaze transactions that are synchronized or attuned. The caregiver synchronizes behavior to the child’s, helping regulate the child’s changing emotions and behaviors, using both verbal and nonverbal expressions, communicating emotions back to the child. Similarly, the National Scientific Council on the Developing Child (2004) described optimal responsive care through the metaphor of “serve and return.” Parents, teachers and other alloparents provide responsive care, initiating and then appropriately responding to infant signals. According to Schore (2009), responsivity involves a wider view of dyadic behavior that involves psychobiological attunement, affective communication between the mother (or caregiver) and infant or child that is bodily based. In non-Westernized contexts, typically, responsive care is provided by a set of caregivers (discussed in the next section) who anticipate the needs of the child and meet them to prevent distress (Gaskins et al., 2017).

#### Responsivity and oxytocin

Responsivity may be the most well studied of the *evolved nest* components regarding effects on oxytocinergic functioning. Often considered the “love hormone” or “bonding hormone,” oxytocin’s role within the parent–child relationship and within responsivity in particular, has garnered years of empirical investigation. This may be due to the fact that parental recognition of their child’s signals is related to brain circuits that belong to a reward system saturated with oxytocin receptors (Wittfoth-Schardt et al., 2012). When fathers view their children’s faces, fMRI scanning shows activation of the ventral striatum and the oxytocin-associated hypothalamus/pituitary region, areas that correspond with reward and attachment. Atzil et al. (2018) found that plasma oxytocin levels were the same for both mothers and fathers when they responded to their infant, highlighting the connection between parental oxytocin levels and the simple task of recognizing or viewing their own child. But there were differences in brain activation locations: in mothers the limbic system was activated whereas in fathers the social-cognitive network was activated.

Other studies have further delineated the connection between oxytocin and parental responsivity by examining synchronous, bio-coregulated behavior. The neuroendocrinological mechanisms that comprise this complex set of interactions are not yet fully known but it is apparent that oxytocin plays a significant role (Szymanska et al., 2017). For example, endogenous oxytocin is known to be associated with increased maternal affectionate behavior, including “motherese” vocalizations, affectionate touch, and expressions of positive affect (Gordon et al., 2010a). For fathers, endogenous oxytocin positively related to stimulatory behavior such as proprioceptive contact, tactile stimulation, and object presentation. Paternal endogenous oxytocin was also associated with father-infant synchrony, especially when fathers played with their infants (Gordon et al., 2010b).

Effects of responsivity on child endogenous oxytocin has also been studied in family situations. Feldman et al. (2010b) examined cross-generational effects by sampling from both parents and their 4–6 months old infants before and after a play interaction. Across both time points, oxytocin levels for both parents and infants increased. However, stronger correlations were associated with higher oxytocin levels in the parent and child dyads that displayed affect synchrony. The research group concluded that oxytocin—and more generally the neuroendocrine system—plays a critical role in supporting bond formation and they highlighted the importance of early experiences in shaping cross-generation transmission of social bonds.

For both parents, endogenous oxytocin has been associated with the degree of physical proximity and affectionate contact with their child. Gordon et al. (2010c) examined both oxytocin and cortisol triadic interactions among infants (6 months), mothers, and fathers. Oxytocin levels in both mothers and fathers predicted higher levels of triadic synchrony, specifically, physical closeness, providing affectionate touch, and coordinating social gaze with the child from one of the parents. For mothers only, higher levels of cortisol were related to lower levels of family synchrony.

Differences in adult attachment styles have been associated with modulation of endogenous oxytocin levels. Attachment quality in adults is a strong positive predictor of oxytocin levels and moderates the relationship between oxytocin and state anxiety (Tops et al., 2007). New mothers who viewed their own 11-month infant smiling and crying showed brain activation differences based upon adult attachment type (Strathearn et al., 2009). Mothers with a secure attachment style showed greater activation in brain reward regions of the ventral striatum and in the oxytocin-associated hypothalamus/pituitary region compared to mothers with an insecure adult attachment style. Plasma oxytocin was also higher in secure mothers. Insecure mothers showed decreased endogenous oxytocin and increased activation in their anterior insula (a region associated with feelings of disgust, pain and unfairness) when viewing their own infant’s face.

Regarding *exogenous* oxytocin, it is associated with changes in paternal parenting behaviors, including alterations to vagal tone. Administration of oxytocin to fathers of 5-month-olds increased paternal vagal tone during free play and facilitated bonding behaviors such as social gaze, affectionate touch, and infant-directed speech (Weisman et al., 2012). Infant oxytocin levels, vagal tone, and social engagement, including social reciprocity, increased in a parallel manner. The results were the first to demonstrate that giving oxytocin to one attachment partner—the father in this case—can induce similar effects in the child, suggesting oxytocin has a potential cross-generational transmission effect through increased social engagement.

#### Responsivity and prosociality

In relation to prosociality, the type of relationship between the parent and child and its influence on oxytocin may be critical. Relationships characterized by a mutual responsive orientation (MRO; Kochanska et al., 1999) are close, mutually binding, cooperative, and affectively positive. MRO between parent and child leads to secure attachment, greater conscience development and prosocial behavior. One can infer that the synchronization that the dyad experiences promotes oxytocin release, facilitating empathy and prosociality. For example, Spinrad and Stifter (2006) investigated maternal responsivity longitudinally, testing infant temperament at 10 months of age in relation to toddler observed empathy at 18 months. Maternal responsivity at 10 months of age predicted higher concerned attention and lower personal distress reactions at 18 months. In another study by Kochanska et al. (1999), maternal responsiveness and shared interactive positivity at 9 and 14 months of age was associated with increased child empathy at 22 months of age. Emotional responsiveness and prosocial tendencies including empathy are correlated in mothers and children (Fabes et al., 1990).

The connection between caregiver responsivity and empathy development makes sense from an evolutionary framework which suggests that responsivity evolved from caregiving behavior rooted in parental investment of offspring (Di Bello et al., 2020). Mothers who are attentive to the changing needs of their infants increase the likelihood that their children develop healthy, regulated physiologies that helps them survive, thrive and reproduce. Thus, mothers and other caregivers would be motivated to sensitively and compassionately recognize and respond to the changing needs of the young offspring. Consequently, adult cooperative sociomoral behaviors would be considered an extension of the caregiving system and intrinsically related to caregiver sensitivity (Swain and Ho, 2017; Gilbert, 2021).

### Alloparents

Allomothers or alloparents refers to multiple, consistently present, responsive caregivers. Within the *evolved nest* context, grandmothers, aunts, siblings, and other related and unrelated kin participate in caring concurrently for the youngest members of the community (“cooperative breeding”; Hrdy, 2009b). Such cooperative child raising, where mother is present but others are caring for the child, increases responsive care from the mother as well as other caregivers who share in the responsibility of meeting the needs of both kin and non-kin children. For example, in a Mayan community, only about half of infant care is typically provided by mothers whereas the other half is provided by other family or community members (Kramer, 2005).

A greater number of alloparents increases the likelihood that children will receive consistent, responsive care (Hrdy, 2009a) and experience longer breastfeeding duration (Quinlan and Quinlan, 2008), improving child adjustment (Pianta and Ball, 1993). When parents are surrounded by a supportive community of alloparents, their knowledge and expectations about parenting increase whereas stress surrounding parenting decreases, allowing for more positive rather than negative interactions with the child (Serrano-Villar et al., 2017). Because alloparents increase wellbeing, they are understood to be a protective factor for both parent and child (Hrdy, 2009a) and may protect against the development of psychopathology (Kenkel et al., 2015). In this way, alloparenting intersects several of the other *evolved nest* components because it involves support for both the parent(s) as well as the developing child.

#### Alloparents and oxytocin

Regarding oxytocinergic functioning, few human studies have examined the role of alloparents. However, there are several animal studies that have examined the role of oxytocin among species who practice alloparenting. For example, among virgin female prairie voles, a cooperative breeding species, alloparent behavior was positively correlated with oxytocin receptor density (Olazabal and Young, 2006) and when given an oxytocin receptor antagonist, adult female alloparent behavior was completely absent. The relationship between oxytocin and alloparenting behavior has also been established in adult male prairie volves (Bales et al., 2004a). In other communal mammals, such as the eusocial naked mole rate, the same association between higher levels of oxytocin receptor density and alloparenting behavior has been observed (Kalamatianos et al., 2010). These studies demonstrate that level of oxytocin receptor density may be a critical determining factor in juvenile and adult mammal alloparenting behavior.

Other animal studies have examined the role of oxytocin as a predictor of future alloparenting behavior. For example, Bales et al. (2004b) found that early exposure to oxytocin, or its antagonist, influences its developmental trajectory and future alloparenting behavior in both adolescence and adulthood. Male offspring given oxytocin *antagonists* on postnatal day 1 had decreased alloparent behavior and increased future attack rates. However, females administered oxytocin (not the antagonist) on postnatal day 1 *increased* alloparenting behavior. These results suggest that oxytocin exposure or deprivation in early life matters for future alloparenting behavior.

Regarding direct effects of alloparenting on human children’s oxytocin, the authors are not aware of any work beyond what has been mentioned for parents. Many non-Western cultures provide a set of consistently present caregivers for the child, offering an area ripe for research.

#### Alloparents and prosociality

Humanity’s heritage of raising children together is associated with psychological changes that enhance social relations such as mind reading and shared intentionality (Burkart et al., 2009). Cross-species studies that include human groups find that extensive alloparental care is the best predictor of variation in proactive prosociality, confirming the origin of humanity’s hyper-cooperation (Burkart et al., 2014).

Laboratory evidence investigating the effect of alloparents on child prosociality is scarce. In a study examining moral socialization in preschool children, the number of kin primary caregivers positively predicted behavior regulation scores when number of primary kin caregivers was less than 4.31, yet negatively predicted scores when the number was greater than 4.31, after controlling for maternal education and household income (Narvaez et al., 2013b). Future research should take into account both number and quality of additional caregivers, whether mother is present during alloparenting, and the effects on children’s prosociality.

### Self-directed free play

Play is a vital part of mammalian childhoods and is shown to promote numerous positive outcomes in children. In nomadic forager communities, humor and playful attitudes infuse daily affairs and activities of all ages, not only through rough-and-tumble play, but in creatively generated riddles, songs, and jokes (Gray, 2011, 2013a). Children are free to explore and play at will. The focus on play is thought to counteract tendencies toward dominance, with other community members deliberately using playful response to quell aggressive and egotistical behaviors (Gray, 2013b; Fry, 2014).

Although play may include organized or structured play, athletic play (sports), pretend play, social play and nonsocial play (Luckey and Fabes, 2005), within the *evolved nest* context, *self-directed free play* is fundamental. Such play promotes affectively beneficial gene expression profiles, emotion regulation, resilience to stress, and may prevent attention-deficit-hyperactivity disorder (ADHD; Panksepp, 2007). The changing dynamics of self-directed free play provides opportunities to learn how to shift and adapt to unexpected actions of playmates, building emotional and relational flexibility (Spinka et al., 2001). A lack of self-directed free play in childhood may contribute to altered social, sexual and conflict interactions with peers (Van den Berg et al., 1999).

#### Play and oxytocin

Self-directed play is a common social behavior and oxytocin’s role in social behavior generally is well established (for a review, see Caldwell, 2017), yet few investigations have examined the role of oxytocin in play. However, there are a few studies that highlight the rise in oxytocin in parents during free play with their children. Feldman et al. (2010a) found that plasma and salivary oxytocin from mothers and fathers increased during play episodes but only for parents who provided high levels of affectionate touch. Social-affective play with their infant but not object-directed stimulatory play increased parental oxytocin in both mothers and fathers (Gordon et al., 2010a). Maternal and paternal oxytocin—measured via plasma, saliva and in urine—were also correlated with the degree of interactive synchrony between parents and their 6-months-old infants during observed play episodes (Feldman et al., 2011). Finally, correlations between parental and infant oxytocin were strongest during play periods when dyads share affect synchrony (Feldman et al., 2010b). However, Markova (2018) failed to find an association between parent-infant play and infant oxytocin levels. Utilizing a sample of 43 mothers and their 4-month-old infants, social game playing was coded for both type and frequency which resulted in 76% of the interactions between the dyads consisted of playful social games and 46 different types of games were identified. Although maternal oxytocin levels were positively associated with the *number* of games played and the *time* spent playing games, infant oxytocin levels demonstrated the *opposite* pattern. Time spent playing games was inversely related to infants’ rise in oxytocin. However, this study did not consider physical proximity or affiliative touch between the dyad, integral aspects of both free play experiences and oxytocin regulation. In addition, the study did not assess dyad synchrony and dys-synchrony.

There is also evidence regarding exogenous oxytocin and fathers’ involvement during play. In a double-blind, placebo-controlled, within-subject designed study, Naber et al. (2010) found that fathers who were administered oxytocin before play sessions with their preschool children were significantly less hostile and more structuring of play to involve the child than in the control condition. This suggests that the fathers were more socially involved and less rigid in their expectations of the play session.

In animal models, the relationship between oxytocin and play is more clearly defined. The reason for the clarity has to do with methodological controls and administration of both oxytocin and its antagonist, experimental conditions that are likely unethical in human studies. For example, blocking oxytocin is known to reduce social play in juvenile rats but only for females and only in certain contexts (Bredewold et al., 2014). Females that are given an oxytocin antagonist show decreased social play but only in new environments. If females are given oxytocin, they also show a decrease in social play behavior but only in the home environment (not novel environment). Males did not show these effects. Taken together, the findings suggests that both context and gender can influence oxytocin’s role in social play behavior.

#### Play and prosociality

In terms of prosociality, by its nature cooperative rough-and-tumble play promotes oxytocin release and empathy (or the playing will stop). For example, Panksepp (2007) demonstrated that play supports the emotion-systems of the brain that give rise to altruism and empathy. Social play accomplishes this by developing the frontal lobe inhibitory skills that underly impulsive emotional urges. Enhancing the development of the frontal cortex promotes abilities to be behaviorally adaptive, self-reflective, and empathic (Panksepp et al., 2003). In this way, social free play builds brains that are socially flexible and relationally attuned to others, skills needed to practice enhanced sociomoral behaviors such as empathy. Indeed, Narvaez et al. (2013b) found that play was associated with greater empathy in preschoolers, even after controls.

Empirical evidence connecting play and prosocial behaviors, such as cooperation, is found in children as young as 2 years of age. For example, Breeland et al. (2022) found that quality of play between unfamiliar 2-year-old dyads predicted subsequent cooperation behaviors. Quality of play was evaluated through affiliation (e.g., helping, directing with a positive tone, encouragement), antagonism (e.g., competing, directing with a negative tone, hitting, neglecting) and dyad coordination. Children who displayed more affiliation during the play period demonstrated greater cooperative motivation in a subsequent novel cooperative task. Quality of play between 2-year-old and *adult* dyads also predicts subsequent child prosociality. In four separate studies, Barragan and Dweck (2014) found that reciprocal play but not parallel play within the dyad related to altruistic behaviors such as helping the adult reach a block, a bottle, a clothespin, and a pencil. The reciprocal play condition included play that engaged the child (such as rolling a ball back and forth) whereas the parallel play condition included the adult playing next to the child with a separate set of toys. The results suggest that reciprocal interactions during play that are characteristic of mutual responsiveness may be strong catalysts for altruism in young children.

### Social embeddedness

Social embeddedness refers to the quantity and quality of connections that surround the individual, both in the family and in the larger environment (Cacioppo and Patrick, 2008). Within nomadic forager communities, relationality and sociality are expansive and the majority of time is spent in social leisure (Gowdy, 1998). In these and village communities, children learn by observing community members and pitching in Rogoff et al. (2015). One aspect integral to social embeddedness observed in nomadic forager communities is a positive emotional climate, an environment that is welcoming and accepting. For children, this means feeling cherished, respected, and appreciated (Tarsha and Narvaez, 2020). In studies with adults, retrospective reports of positive childhood home climate were correlated with higher scores on secure attachment and mental health, whereas negative home climate experiences were associated with low scores on secure attachment and higher scores on anxiety and depression (Narvaez et al., 2016b,c).

#### Social embeddedness and oxytocin

The construct of social embeddedness and its relationship to endogenous oxytocin is largely understudied, although social support and oxytocin release interact to decrease physiological and psychological stress (Heinrichs et al., 2003). Studies comparing children from orphanages to children under typical conditions demonstrate that the buffering effect of oxytocin and social support are dependent upon early experience. Unlike birth children, adopted children do not show an increase in oxytocin levels after playing a physical contact game with their mothers (Wismer Fries et al., 2005). Further, birth children had a decrease in cortisol whereas the adopted children showed an increase, a response that is comparable to engaging with a stranger. These findings suggest that the social buffering effects of oxytocin may be dependent upon the early relationship with a responsive caregiver and that individuals with an unsupportive early life may not derive the same stress relief and oxytocin rise from social support (Hostinar and Gunnar, 2015). Stated succinctly, the effectiveness of the social buffering of stress through oxytocin may be dependent upon other *evolved nest* components, such as positive touch and responsive caregiving.

Regarding *exogenous* oxytocin, several studies demonstrate a connection between experiences of social support and oxytocin. For adults, several studies found that administering oxytocin intranasally may increase the effect of social support and even change attachment styles. For example, in adult males who were categorized as insecurely attached, a single dose of intranasal oxytocin significantly increased their security level (attachment style; Buchheim et al., 2009). Oxytocin in men also enhanced the effect of social support (presence of their best friend) during a stress condition. Exogenous oxytocin also decreased men’s cortisol and anxiety levels significantly, boosting their sense of calmness when their friend was present (Heinrichs et al., 2003). As such, these findings, in addition to other empirical studies with women (Ozbay et al., 2007; McQuaid et al., 2016), suggest that perceived social support and oxytocin may be intrinsically linked.

#### Social embeddedness and prosociality

Regarding prosociality, to the authors’ knowledge, there are no studies that examine physiological correlates of social embeddedness, especially oxytocin, and its relation to empathy and prosociality across development. More research is needed that examines the relation among these constructs. However, social exclusion is shown to decrease prosocial behavior (decreased helpfulness, cooperation, volunteering, donating) in laboratory studies (e.g., Twenge et al., 2007). There is also some evidence that social support can push adults toward pro- or anti-sociality (against outgroups; Declerck et al., 2010; De Dreu et al., 2011). Oxytocin is also sometimes associated with antisocial behavior and aggression (DeWall et al., 2014). For example, both endogenous and exogenous oxytocin’s effects on sociomoral behavior are influenced by complex interactions involving social embeddedness and group identity (Berry, 2013). These sociomoral outcomes include increased outgroup discrimination and reduced affiliative behavior following exogenous oxytocin administration. However, oxytocin’s antisocial effects are not associated with unprovoked hateful intergroup behavior (when administered intranasally; De Dreu et al., 2011). As such, oxytocin’s effects on strengthening bonds may be one aspect of its influence on prosociality, especially when considering social groups and group identity.

### Nature immersion

In our ancestral context, human communities were immersed in and attached to the rest of the natural world from an early age, feeling a part of nature, observing animals and plants, enabling awareness of how to promote wellbeing in the biocommunity (Kimmerer, 2013). Several scholars have pointed to the lack of ecological attachment—emotional connection—to the natural world as a factor contributing to human conflict and our current ecological crises (Louv, 2005; Berry, 2013). Because more than half of the global population lives in urban areas, research regarding the effects of nature experiences is increasing (Mayer and Frantz, 2004; Matthew et al., 2009; Ives et al., 2017). Several population-based studies found that nature exposure or frequency of interacting with a green space increased positive emotions and general wellbeing as well as reduced stress and possibly even morbidity and mortality (Cleary et al., 2017).

#### Nature immersion and oxytocin

Like many of the *evolved nest* components, nature immersion promotes calmness, lowering cortisol, improving immune function (Frumkin et al., 2017; Bratman et al., 2019). However, there is a dearth of research examining one of the contributors to all these factors, oxytocin release. To the authors’ knowledge, no studies have examined nature immersion and oxytocinergic functioning, suggesting more research is needed in this area.

#### Nature immersion and prosociality

Nature immersion promotes prosociality such as generosity, ethical decision making, prosocial values (Piff et al., 2015) and cooperation (Zelenski et al., 2015). In a within-subjects design with elementary school students, Dopko et al. (2019) compared children after experiences at a forest and nature school with experiences at an aviation and space museum. They compared not only positive/negative emotion, connection to nature and its protection but prosocial behaviors. Students behaved more prosocially after the nature school experience. More recently, Putra et al. (2020) conducted a systematic review of evidence relating green space and child prosocial behaviors. Utilizing 15 studies, 44 positive associations were identified of which 18 were statistically significant, suggesting that green space may augment prosocial behavior in children and adolescents. However, given variations in green space exposure as well as a lack of consideration of moderators, evidence for the association as well as causality should be interpreted as preliminary. This area of study is ripe for research.

### The direct path between the *evolved nest* and prosociality

Apart from physiological effects, there are emerging connections for both children and adults regarding *evolved-nest* consistency experience and prosociality generally. The *evolved nest* enables individuals to develop rich social connections that catalyze personal growth, thriving, and virtue across the lifespan (Narvaez, 2016). Such capacities support societal functioning at large, enabling cooperative interactions in families (Lomanowska et al., 2017) and communities (Narvaez, 2014). As such, evidence from both animal and human studies suggest connections between *evolved nest* experience and prosociality without taking into account oxytocin functioning (Figure 1, path c). For example, in mice, communal rearing and alloparents are known to increase the amount of maternal care the offspring receives which is highly correlated with increased sociality (Branchi et al., 2006; Curley et al., 2009). There are also observed differences regarding communal rearing and sociality in prairie voles. Compared to those raised by a single mother, prairie vole offspring in the communal reared environments show increased social interactions, higher social competency and form social hierarchies quicker (Kenkel et al., 2015). The increased sociality is seen with both peers and nonpeers, effects evident into adulthood. This suggests that for social mammals communal rearing facilitates an increased ability to engage and interact appropriately with differing environments and contexts (Branchi et al., 2013).

Regarding human studies, there are connections emerging regarding *evolved nest* experience and prosociality in adulthood. For example, adults (*N* = 606) reported on *evolved nest* experience in childhood and sociomoral capacities of perspective taking and empathy as well as sociomoral orientations of engagement, social opposition, and social withdrawal (Narvaez et al., 2016c). Mediation analysis demonstrated that early childhood experiences consistent with the *evolved nest* related to greater perspective taking and lower social opposition as well as lower personal distress and lower social withdrawal. Mediators included secure adult attachment and lower psychopathology. In this sample, mediation analysis did not explain empathy by way of *evolved nest* experience, which the authors suggest may be attributed to the influence of psychopathology, specifically, depression, which can increase empathy. In a corroborating study, *evolved nest* history followed similar pathways (Narvaez et al., 2016a). Those with greater nestedness or experiences that aligned with the *evolved nest*, had higher secure attachment, better mental health and perspective taking which led to greater social engagement. Those with less nestedness either followed a pathway of lower secure attachment, low mental health, and low perspective taking leading to social oppositionalism or a pathway with low secure attachment, low mental health, high personal distress and social withdrawal in social situations.

There are also connections between *evolved-nest* experience and childhood prosociality. In a 3-year longitudinal (prenatal to age 3) study of mother–child dyads at risk for child neglect using observation and interview, *evolved nest* experience longitudinally predicted increased child prosociality at 18 and 30 months of age (Narvaez et al., 2013a).

Taken together, the evidence suggests that each *evolved nest* component may support capacities for empathy and prosociality. Evidence suggests that a mediator between *evolved nest* experience and capacities for empathy and prosociality may be oxytocinergic functioning.

## Discussion

Our review addresses a major gap within the literature regarding investigations that examine all three constructs of early life *evolved nest* experience, oxytocinergic and sociomoral functioning. We examined what is known about how each *evolved nest* component—soothing perinatal experiences, breastfeeding, touch, responsivity, allomothers, play, social embeddedness, and nature connection—is associated with oxytocinergic functioning and sociomorality, specifically, empathy and prosociality. Across studies that were available, *evolved nest* provision was associated with increased *endogenous* oxytocinergic functioning and positive sociomoral outcomes in both children and adults. *Exogenous* oxytocin was associated with *evolved nest* provision behaviors, though with mixed results concerning perinatal experiences. That is, exogenous oxytocin administered to the mother during the perinatal period has potential adverse effects, decreasing the probability of bonding and breastfeeding duration.

Availability of empirical investigations varied for the triumvirate focus (evolved nest, oxytocin, prosociality). Most studies addressed responsivity whereas few studies examined social embeddedness and nature immersion. Of the few studies that include all three constructs, most focus on *negative* early life experiences and their influence on oxytocin and sociomoral development. For example, Kompier et al. (2019) conduced a review examining unnested experience or experiences that are antithetical to the *evolved nest* (early life stress of maternal separation, paternal deprivation, and postnatal isolation) on adult social behavior and oxytocin and vasopressin. Integrating causal animal studies, the researchers found that early life stress significantly impacted the oxytocinergic system in brain regions associated with social behavior and suggested similar connections in humans. Their review highlights the role of negative early life experiences in altering both oxytocinergic functioning and adult sociality. Our review flipped the perspective, examining the importance of *positive* early life experiences of the *evolved nest* that may optimize physiological and sociomoral outcomes.

Our review also highlights possible interaction effects that should be investigated. First, there may be interactions among *evolved nest* components themselves. For example, during breastfeeding (one *evolved nest* component), the infant is bathed in numerous sensory experiences that include other *evolved nest* components such as touch and maternal responsiveness (vocalizations and eye contact). How long the infant is breastfed may be influenced by how much exogenous oxytocin was given to his/her mother during childbirth (Gu et al., 2016), another *evolved nest* component. In this way, *evolved nest* components of perinatal history, touch, breastfeeding, and maternal responsivity interact, possibly exponentiating effects on child physiological and sociomoral development. Second, there may be complex interactions between parental physiological processes and prosocial, empathic parenting behaviors. Nurturing parenting behavior is dependent upon healthy physiological processes that co-regulate the development of child physiological functioning and pro-social behaviors. For example, a recent review by Froemke and Young (2021) demonstrated that virgin female rats’ exposure to maternal behavior increased their oxytocin responses. Housed with other mothers, virgin females’ pattern of oxytocin release changed to match the experienced mothers when they heard infants crying. This demonstrates the powerful impact of the social environment on shaping *evolved nest* provision and oxytocin responses, at least in animal models. Thus, the interaction and directionality among these three constructs (*evolved nest*, oxytocinergic functioning and prosociality) appears to be more complex than direct linear associations and may include exponentiating, mediating, and moderating effects. In summary, there is emerging evidence that each *evolved nest* component may contain complex, bidirectional interaction effects both between *evolved nest* components themselves as well as among physiological and sociomoral processes. When *evolved nest* experience is provided in full, it represents a type of ecological context that can be understood as childhood companionship care or experiences that support the biology of love.

### Is the *evolved nest* integral to a biology of love?

The ecological context of the evolved nest may be critical for humanity’s species-typical moral development because our morality is rooted in our neurobiology (Narvaez, 2017). The *evolved nest* represents part of what biologist Humberto Maturana calls the *biology of love*, childhood companionship care that is critical for species normal development (Maturana and Verden-Zöller, 2008). Maturana argues that humans are the kind of biological beings they are because love has grounded the course of evolutionary history that originated the species, which he calls *homo sapiens-amans*. He says that animals generally, but mammals particularly, have two modes of being: mutual relational trust or dominance hierarchy. In other species, the former characterizes mother-offspring relations and the latter adult relations. In contrast, the human lineage maintained the relational dynamics of mother–child love into adulthood (called neoteny), like trust, tenderness, sensuality, and playfulness. “Humans are cooperative animals dependent on love at all ages” (Maturana and Verden-Zoller, 2008, p. 54) and “become ill at any age if we have to live a life centered in mistrust, instrumentalization, and manipulation of relations” (Maturana and Verden-Zoller, 2008, p. 52).

*Evolved nest* provision in early life may represent the biology of *love in action*. *Evolved nest* provision communicates that the child matters, that their needs are legitimate as a fellow being. Experiencing the *evolved nest* may marinate the child in a positive biochemistry that supports the healthy development of neurobiological systems, such as the oxytocin system. Concurrently, the child builds psychosocial memories and skills for living, including getting along with others. Experiences of love in action starting in the perinatal period provide the child the ecological context that optimizes physiological and psychological processes needed to learn how to love (Carter, 2022). This includes behavior interactions associated with love such as emotional safety and healthy sexuality.

The oxytocinergic system may be a critical feature of emotional safety and healthy sexuality, or the biology of love, providing “sociostasis” throughout life (Carter, 2022). Sociostasis refers to the influence each person has on the other in terms of regulating the other person’s physiology, psychology, and state of mind (Cozolino, 2014). Carter employs a wider use of the term to capture the importance of sociality regarding both homeostatic and allostatic processes of physiology and behavior. The central role of oxytocin in sociostasis may be attributed to its role in modulating reactions to stress after experiences of challenge by interacting with the HPA axis to calm the organism, reducing inflammation, and anticipating future allostatic demands (Carter, 2021). Oxytocin’s anti-stress properties may facilitate prosociality because of its role in maintaining allostasis which undergirds sociostasis (Carter et al., 2020).

Maturana expressed concern that the biology of love has been forsaken by the dominant culture, undermining the development of *homo sapiens-amans* and instead shifting human evolution toward *homo sapiens-aggressans and homo sapiens-arrogans* because “the manner of living conserved from one generation to the next, as a particular configuration of organism-niche relations, becomes the operational dynamic center around which everything else is open to change” (Maturana and Verden-Zoller, 2008, p. 2). As one of humanity’s extra-genetic inheritances, along with cells, body plans, ecology, and culture (Oyama et al., 2001; Jablonka and Lamb, 2005), the *evolved nest* may be critical for shaping the biology that undergirds health through sociostasis (Carter, 2022) and the biology that shapes culture. If attachment is a regulatory theory (Schore, 2003) that fosters various forms of self-regulation in the child, the *evolved nest* may provide the mechanism by which regulation occurs, affecting multiple systems including the oxytocinergic system.

Given the importance of sociostasis and oxytocin functioning, the most critical aspect of *evolved nest*-oxytocin interactions may be social relationships. For example, endogenous oxytocin release and exogenous oxytocin administration demonstrate varying effects dependent upon relationship, specifically, the social context yielding either evocation of affiliation or anxiety. This suggests that the quality and type of relationship matters for what neurobiological reactions occur. With affiliation and feelings of safety, oxytocin and the social brain network are activated. With a feeling of unsafety or threat, the HPA axis will be activated, disrupting oxytocin release (Carter, 2022).

## Limitations

There are multiple limitations for this investigation. We did not address epigenetics, such as the methylation of the oxytocin receptor gene. Caregiver behavior is associated with epigenetic manipulations of the oxytocinergic system in early childhood (e.g., Krol et al., 2019).

In terms of empirical evidence reviewed, the separation of variables from context is problematic. Ethnographic studies that describe the *evolved nest* in nomadic forager communities are ecological contexts where *all* of the *evolved nest* components are provided simultaneously and generously. In contrast, empirical studies use variable measures and do not measure amount or frequency in the *evolved nest* manner (e.g., breastfeeding on request; holding and carrying in arms). Thus, when comparing findings from empirical studies and ethnographic observations, there may be major differences in frequency or exposure of each *evolved nest* component. In our review, studies that controlled specific amounts or deprivation of a particular *evolved nest* component were animal studies (more touch vs. less touch, low responsivity vs. high responsivity), though it is not always clear whether the component was isolated from other component effects. Conducting similar studies with human samples may be too onerous and if involving negative treatment, unethical. Moreover, when the *evolved nest* is provided in full, this may influence neurobiological and sociomoral development in ways that are beyond the summation of its parts given that multidimensional, complex interactions are taking place both behaviorally and neurobiologically.

We only examined empathy and its correlates. There are multiple other sociomoral outcomes that could be examined. For example, Narvaez et al. (2013b) found that after controls; breastfeeding was related to preschoolers’ inhibitory control, guilt and concern after wrongdoing; positive touch was related to inhibitory control, self-regulation and concern after wrongdoing (as well as empathy), family social embeddedness and play to inhibitory control; self-regulation and concern after wrongdoing were associated.

Finally, there were limitations regarding the empirical evidence reviewed and developmental processes of timing. By definition, the *evolved nest* is the ecological system of care that aligns with the child’s maturational schedule: each *evolved nest* component is provided in such a way that it supports the child’s varying physical and psychological needs (e.g., abundant touch in the first year of life through carrying, rocking vs. affectionate touch of hugs and hand holding later in development). The empirical evidence reviewed largely does not take into account the developmental timing of each *evolved nest* component. Moreover, variations exist within each *evolved nest* component regarding intensity, duration and exposure needed to support optimal child development and enhance both the oxytocinergic system and sociomoral development.

## Future directions

There are many opportunities for future work knitting together early experience, particularly experiences reflective of the *evolved nest*, oxytocinergic development, and prosociality. Whereas the construct of oxytocin has become more precise (Carter, 2022), the definitions and constructs of *evolved nest* provision are less established. The eight components of the *evolved nest* are descriptors garnered from observations of humanity’s evolved context. But likely additional components could be extracted such as agency/autonomy and embedded relations with wild plants and animals. Moreover, we need to study contextualized *evolved nest* components, such as *moving* touch for its obvious benefits (e.g., Mason and Berkson, 1975; Ohmura et al., 2022).

Furthermore, several authors associate and mix sociomoral behaviors with parenting behaviors, often considering nurturing responsive care and prosocial behavior, compassion, and empathy as synonymous. For example, nurturing responsive care is sometimes viewed as containing elements of sociomoral behaviors where nurturing care *is* compassionate parenting; nurturing care *is* empathic; nurturing care *is* prosocial (Saturn, 2017; Swain and Ho, 2017). Often in the literature these constructs are interchangeable or interwoven. This evokes the need for a general consensus regarding constructs and definitions of both parenting behaviors and their relationship to prosociality. Future research should consider ways to clarify and operationalize both evolved nest provision and sociomoral constructs.

## Conclusion

Research into sociomoral development typically focuses on parent socialization practices such as verbal guidance and discipline, as if an unruly body must be sorted out by the rational mind of the adult until the child’s own rationality is in place. If biology is addressed, it usually focuses on genes and built-in temperament, as if children are born a particular way. The sciences have advanced us beyond these limited perspectives. The central core of development is children’s psychobiological self-organization as an interaction between in-built maturational schedules and experience (Maturana and Varela, 1980; Sansom and Brandon, 2007). The interactions of external and internal signals are constant in early life as the brain grows rapidly, building synapses and pruning them, based on experience in a type of contingent “constructive interactionism” (Oyama, 2000; Oyama et al., 2001), forming a life cycle. Only when we integrate biology, psychology and evolution in a holistic manner, will we be able to discern the species-normal course of sociomoral development (Narvaez, 2014). Thus, investigations into prosociality need to start in the relational contexts and the contingencies a child experiences that build the body–mind (Narvaez et al., 2022). The most sensible framework for examining what builds a healthy life cycle and optimizes prosociality may be the millions-year-old *evolved nest*.

## Author contributions

All authors listed have made a substantial, direct, and intellectual contribution to the work and approved it for publication.

## Conflict of interest

The authors declare that the research was conducted in the absence of any commercial or financial relationships that could be construed as a potential conflict of interest.

## Publisher’s note

All claims expressed in this article are solely those of the authors and do not necessarily represent those of their affiliated organizations, or those of the publisher, the editors and the reviewers. Any product that may be evaluated in this article, or claim that may be made by its manufacturer, is not guaranteed or endorsed by the publisher.
